# Transcriptome analysis of postharvest blueberries (*Vaccinium corymbosum* ‘Duke’) in response to cold stress

**DOI:** 10.1186/s12870-020-2281-1

**Published:** 2020-02-19

**Authors:** Fan Zhang, Shujuan Ji, Baodong Wei, Shunchang Cheng, Yajuan Wang, Jia Hao, Siyao Wang, Qian Zhou

**Affiliations:** College of Food, Shenyang Agricultural University, No.120 Dongling Road, Shenhe District, Shenyang City, Liaoning Province 110866 People’s Republic of China

**Keywords:** Blueberry, Differentially expressed genes, Low temperature storage, Pathways, Pitting, Transcriptome analysis

## Abstract

**Background:**

Blueberry (*Vaccinium spp.*) is a small berry with high economic value. Although cold storage can extend the storage time of blueberry to more than 60 days, it leads to chilling injury (CI) displaying as pedicle pits; and the samples of 0 °C-30 days was the critical point of CI. However, little is known about the mechanism and the molecular basis response to cold stress in blueberry have not been explained definitely. To comprehensively reveal the CI mechanisms in response to cold stress, we performed high-throughput RNA Seq analysis to investigate the gene regulation network in 0d (control) and 30d chilled blueberry. At the same time, the pitting and decay rate, electrolyte leakage (EL), malondialdehyde (MDA) proline content and GSH content were measured.

**Results:**

Two cDNA libraries from 0d (control) and 30d chilled samples were constructed and sequenced, generating a total of 35,060 unigenes with an N50 length of 1348 bp. Of these, 1852 were differentially expressed, with 1167 upregulated and 685 downregulated. Forty-five cold-induced transcription factor (TF) families containing 1023 TFs were identified. The DEGs indicated biological processes such as stress responses; cell wall metabolism; abscisic acid, gibberellin, membrane lipid, energy metabolism, cellular components, and molecular functions were significantly responsed to cold storage. The transcriptional level of 40 DEGs were verified by qRT-PCR.

**Conclusions:**

The postharvest cold storage leads serious CI in blueberry, which substantially decreases the quality, storability and consumer acceptance. The MDA content, proline content, EL increased and the GSH content decreased in this chilled process. The biological processes such as stress responses, hormone metabolic processes were significantly affected by CI. Overall, the results obtained here are valuable for preventing CI under cold storage and could help to perfect the lack of the genetic information of non-model plant species.

## Background

Both production and consumption of blueberry (*Vaccinium spp.*) have increased sharply worldwide in recent years at least partly due to their known nutritional, economic value and health benefits. However, it can only be stored for 5–10 days at room temperature (RT) due to the rapid softening. Since how to prevent the decay and prolonging the shelf-life became important topics in postharvest research. Storage at low temperature (LT) is advantageous to preventing softening and prolonging the postharvest life; however, cold storage triggers the pedicle pitting, pericarp and pulp adhesion in postharvest blueberry; which also accompanied with abnormal changes of stress-related enzymes, destruction of outer cell wall structure, and the decrease of fiber filaments [1, 2]. The development of this CI reduces consumer acceptance of the fruit, thus limiting its storage life. And it has been confirmed that cold stress accelerated the energy consumption, membrane lipid peroxidation and affected normal active oxygen metabolism in blueberry.

Chilling injury is one of the main problems affecting the market value of horticultural fruits and vegetables; which is also one of the principal abiotic stress. Cold stress at low temperatures above zero (0–10 °C) and freezing stress at subzero temperatures lead to decreased cell membrane fluidity, water potential, osmotic stress causing irreversible damage. In previous research, cold stress (4 °C) has been found to damage the cellular structure of peppers (*Capsicum annuum L.*) [3–5]; and the most typical CI symptom in peppers is water immersion sunken spots [6, 7]; in banana, the peel would pit and discolor at low temperatures [8]; in peach, CI symptoms include lack of juiciness [9], poor flavor, and the brown-red discoloration of flesh [10]; in mango, CI symptoms include scalding, softening, internal browning and electrolyte leakage [11]; in litchi, fruit quality, membrane permeability, enzyme activities and energy changed during cold storage [12]; in pear, the aroma was less under or after long term storage at 0 °C, seriously affecting its quality [13–15]; in harvested cucumber (*Cucumis sativus L.*) fruit, disorder characterized by surface pitting and dark watery patches was confirmed when when held at 7 °C or below [16]. In conclusion, it has been identified when the fruits are subjected to cold stress, the cell structure changed, the membranes disrupted, the malondialdehyde (MDA) content increased, and the reactive oxygen (ROS) accumulated [17, 18], finally led to the decline of fruit quality. Although the CI symptoms in different fruits and vegetables are distinct, the nutritional and economic values are adversely affected. And the mechanisms involved in the CI of blueberry are still not clear. Therefore there also remains a need to study the chilling mechanism and develop more effective techniques for blueberry fruit storage and transport at LT. Moreover, with the rapid development of high-throughput sequencing, great progress has been made towards the understanding the cold-response mechanism underlying CI.

Among these, RNA-sequencing (RNA-Seq) has become a powerful technology for characterizing molecular regulators in numerous postharvest fruits, such as peppers, tomatoes, and ‘Nanguo’ pear [14, 19]. Additionally, (Zhao et al. 2019) [20] identified four PbBAM genes associated with stress in pear using RNA-Seq; (Lou et al. 2018) [21] combined the isobaric tags for relative and absolute quantification (iTRAQ) and RNA-Seq illustrated the mechanism of cold tolerance in the loquat. Previous studies have also identified and confirmed the expression pattern of some key genes and transcription factor (TFs) are in response to cold stress. Among these, bZIP, MYB, CBF, and AP2/ERF are the most well-known TFs and are regarded as important regulators of cold-responsive genes in plants [22–28]. However, CI studies on postharvest blueberry are scarce and the genetic characterization of the underlying regulatory mechanisms of pitting in blueberry has rarely been investigated.

In the present study, in order to characterize the CI mechanism at the molecular level, enrich available transcriptome data, identify genes, important pathways and regulation network potentially involved in fruit response to cold storage, a comprehensive transcriptome profiling analysis and physiological experiments were performed on 0d (control) and 30d chilled blueberry (pitted). The results obtained here provide a theoretical and a practical basis for preventing blueberry pitting; it is also the basis for studying the functions of genes and transcription factors affecting the blueberry fruit pitting and provide a reference transcriptome for future studies involving blueberry fruit.

## Results

### Changes in phenotype and physiology at low temperature

#### Changes in blueberry fruit phenotype at low temperature

**Fig. 1:**
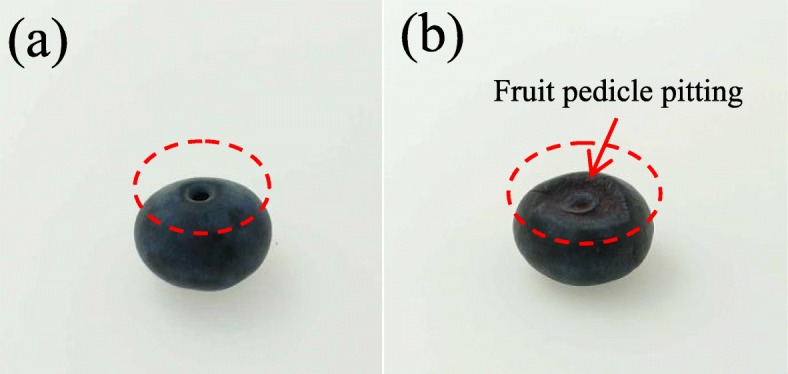
The appearance of blueberry (**a**) without pitting, (**b**) with pitting, 30d chilled berries

The physiological performance of control blueberry and 30d chilled blueberry was characterized. The blueberry stored at 20 °C did not show CI symptoms (Fig. 1a), whereas the fruits stored at 0 °C for 30 days showed typical CI symptoms as pedicle pitting (Fig. 1b); these symptoms were maintained during the shelf-life.

#### Changes in pitting rate and decaying rate at low temperature

**Fig. 2:**
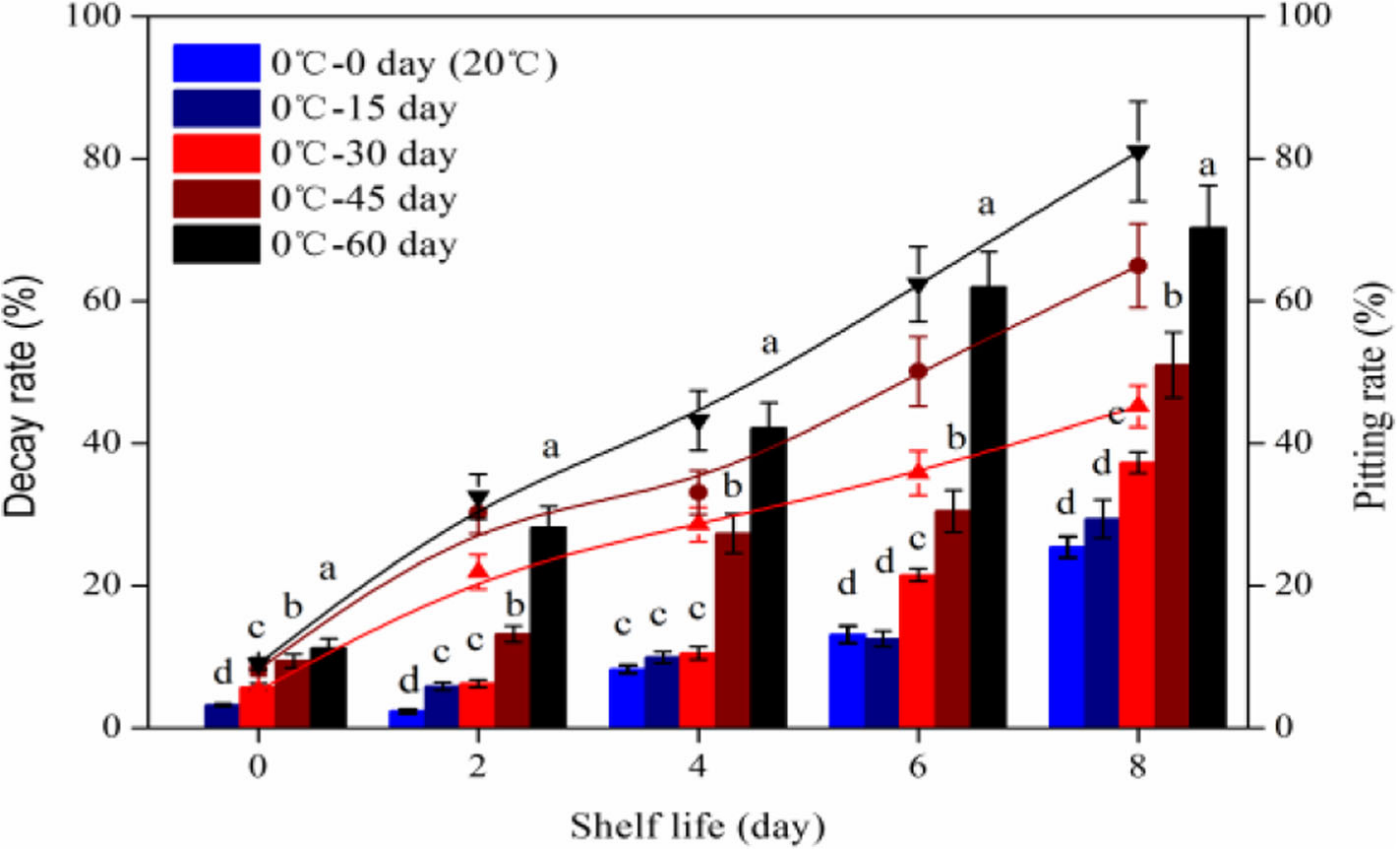
Changes of pitting and decay rates at shelf-life (20 °C) after different storage time at 0 °C. The lines indicate the pitting rate and the column diagram indicate the decay rate. Each value is the mean of three replicates of 150 fruits (*P* < 0.05). The error bars show the SD of the means (*n* = 3; three biological replicates, each with three technical replicates)

After the measurements of the pitting rate in blueberry at shelf-life after different storage times at 0 °C; we found no signs of pitting were observed in the blueberry stored directly at 20 °C or for those stored at 0 °C for 15 days beforehand (Fig. 2). However, signs of pitting were observed in blueberry stored at 0 °C for 30 days beforehand. The pitting rate was 5.2% after 30 days 0 °C storage, after then rapidly increased to 21.9% at 2d shelf-life, and then 28.6% at 4d, 35.8% at 6d and 45.2% at 8d. For blueberry stored at 0 °C for 45 days and 60 days, the pitting rates increased to 65.3 and 81% when the fruits were removed from 0 °C and kept at 8d shelf-life. The decay rate had a similar trend; both increased gradually, but the decay rate was slightly higher in long-term low temperature stored blueberry than that in the short-term low temperature stored (Fig. 2).

#### Changes in physiology at low temperature

**Fig. 3:**
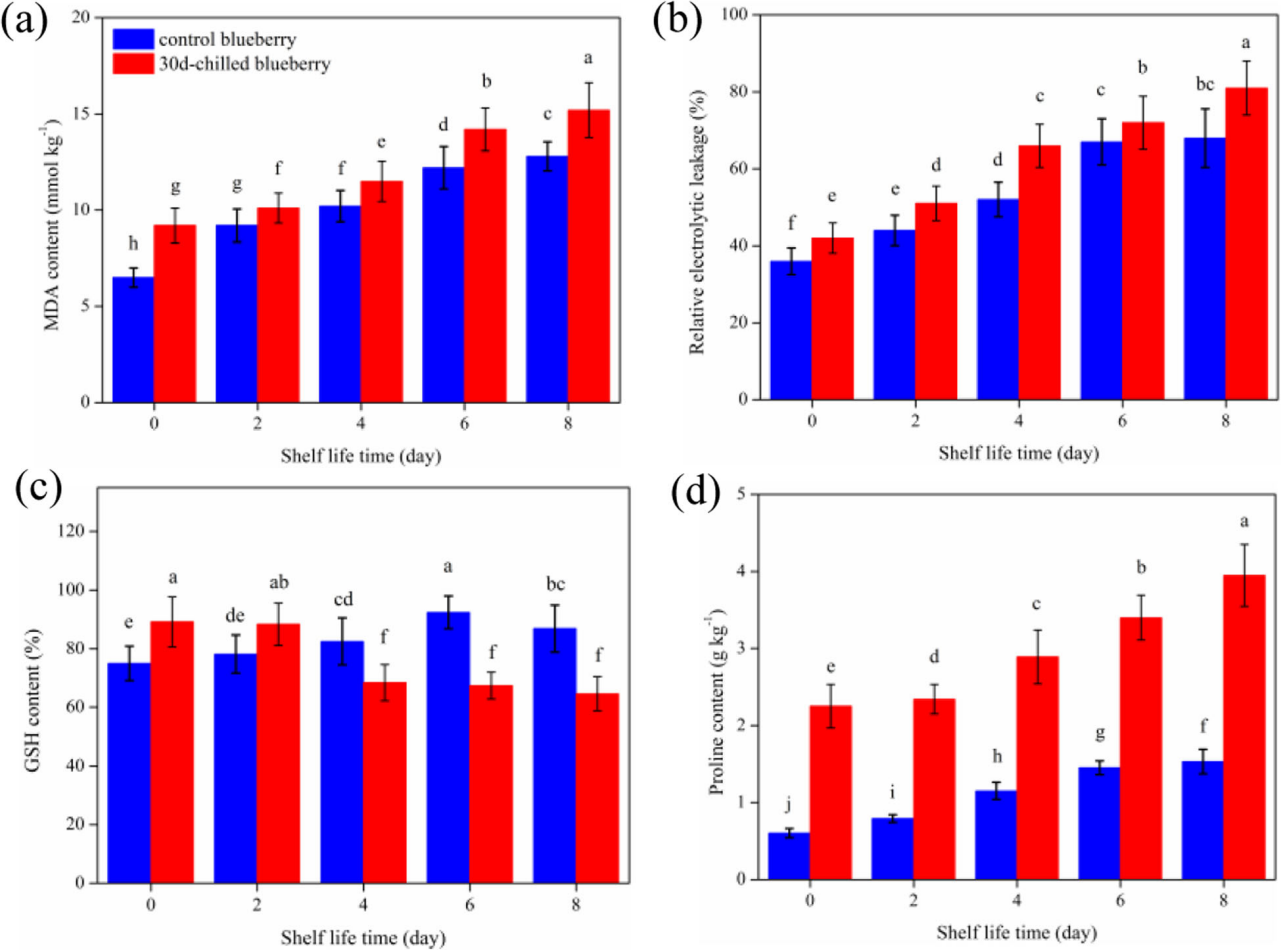
Changes in (**a**) MDA content, (**b**) relative electrolyte leakage (EL), (**c**) GSH content, (**d**) proline content at shelf-life and shelf-life after 30 days’ 0 °C storage. The error bars show the SD of the means (n = 3; three biological replicates, each with three technical replicates)

Membrane plays a key role in fruit. Membrane lipid peroxidation often occurs during CI and MDA is one of the products of membrane lipid peroxidation. As shown in (Fig. 3a), there was an upward trend in the MDA content, and the MDA content of blueberry fruit stored at 0 °C for 30 days (9.2 mmol kg^− 1^) was higher than that of fruit stored at 20 °C (6.5 mmol kg^− 1^). Meanwhile, the permeability of the cell membrane can reflect the stability and the injury degree of the cell membrane; which can reflect by the cell membrane relative conductivity. It can be seen from (Fig. 3b) that with the prolongation of storage, the permeability of cell membrane of blueberry fruit increased continuously. After 30 days storage at 0 °C, membrane structure, phase change and permeability changed; the relative electrolytic leakage of the blueberry stored at 0 °C for 30 days (42%) was higher than that of blueberry stored at 20 °C (36%), then reached to 81%. Meanwhile, with the occurrence of pedicle pitting, the relative conductivity of fruit cell membrane increased sharply. The results showed that the GSH content in blueberry fruit stored at 0 °C for 30 days (89.2%) was higher than that of fruit stored at 20 °C (75%); the GSH content accumulated with the prolongation of time, especially in the fruits stored at 20 °C-6 days (92.4%) (Fig. 3c). After 30 days’ storage at 0 °C, the GSH content of blueberry fruit decreased rapidly from 89.2%, and then maintained a low stable state (64.6%). The proline content of blueberry fruit stored at 0 °C for 30 days (2.25 g kg^− 1^) was higher than that of fruit stored at 20 °C (0.6 g kg^− 1^) (Fig. 3d), and the proline content increased significantly after the fruits were removed from cold storage and reached 3.95 g kg^− 1^ at 8d shelf-life. The results indicated that the cell membrane structure and membrane function were seriously damaged during cold storage.

### Summary of the sequencing data

**Fig. 4:**
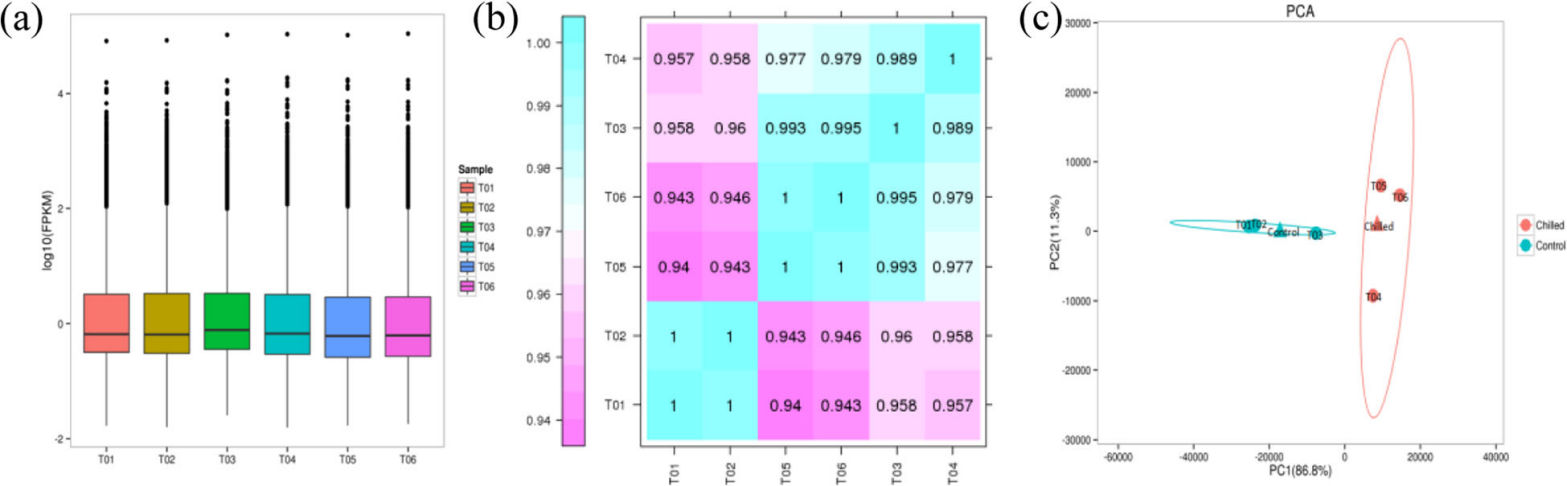
(**a**) Box plot representation of expression range from six libraries; (**b**) Pearson correlation between samples and (**c**) PCA analysis among T01-T06 samples; Different colored squares represent the degree of correlation between two samples; T01-T03 represented 0d control samples and T04-T06 represented 30d-chilled samples

To study the effect of cold storage on postharvest blueberries, total RNAs from six samples (control and chilled) were used for deep sequencing. To evaluate the quality of the sequencing data, randomization test of mRNA fragmentation saturation, length test of insert fragment and saturation test were conducted. These tests determined the number of obtained reads was enough to cover most of the expressed genes. The total 50.74Gb of clean data were generated (Table 1). Average clean data were above 6.25Gb and Q30 values were obtained for more than 93.93% of the data; 76.38 to 79.53% of the clean reads were successfully mapped to the unigene database. Meanwhile the transcription group data detection has a high sensitivity, with the protein encoding gene expression level FPKM (fragments per kilobase million) having values across 10^− 2^ to 10^− 4^ six orders of magnitude. The dispersion degree of sample gene expression level distribution was average, and the overall gene expression abundance in different samples was good (Fig. 4a). According to the PCA analysis, the three biological replicates of control and chilled groups were clustered together respectively (Fig. 4c), showing a Pearson correlation above 0.94 (Fig. 4b).

**Table 1:** Summary of RNA-Seq data and sequence assembly

Samples	Base Number	Clean Data			Error(%)	% ≥ Q30
		Clean Read	Mapped Reads	Mapped Ratio		
T01	9,135,928,834	30,590,963	23,364,104	76.38%	0.01	94.43%
T02	9,105,416,678	30,487,303	23,308,114	76.45%	0.01	93.93%
T03	6,252,371,304	20,941,667	160,042,397	76.61%	0.01	94.14%
T04	8,567,949,538	28,664,023	22,285,082	77.75%	0.01	94.29%
T05	9,096,999,984	30,453,155	24,218,167	79.53%	0.01	94.25%
T06	8,580,914,322	28,746,121	22,703,600	78.98%	0.01	94.79%

Note: Read Number: Total pair-end reads in the clean data; Base Number: Total base number in the clean data; % ≥ Q30: Percentage of bases whose clean data mass value is greater than or equal to 30

### Comprehensive profiling of transcript expression analysis

#### Number of DEGs

**Fig. 5:**
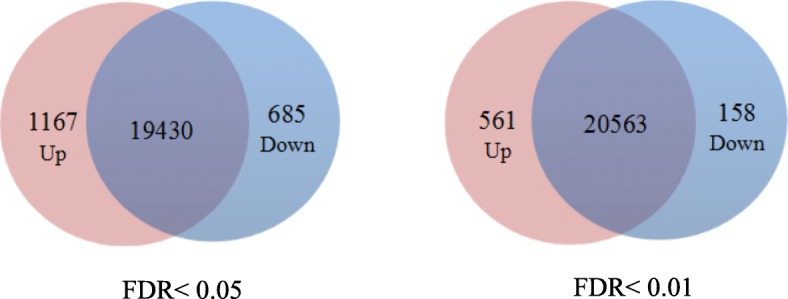
Venn diagram of differentially expressed genes between each two samples

The analysis of DEGs revealed 561 up-regulated and 158 down-regulated genes for FDR ≤ 0.01, and 1167 up-regulated and 685 down-regulated genes for FDR < 0.05. To assess the diversity of DEGs, the Venn map was constructed (Fig. 5).

#### Heatmap of all DEGs and the selection of significantly changed DEGs

**Fig. 6:**
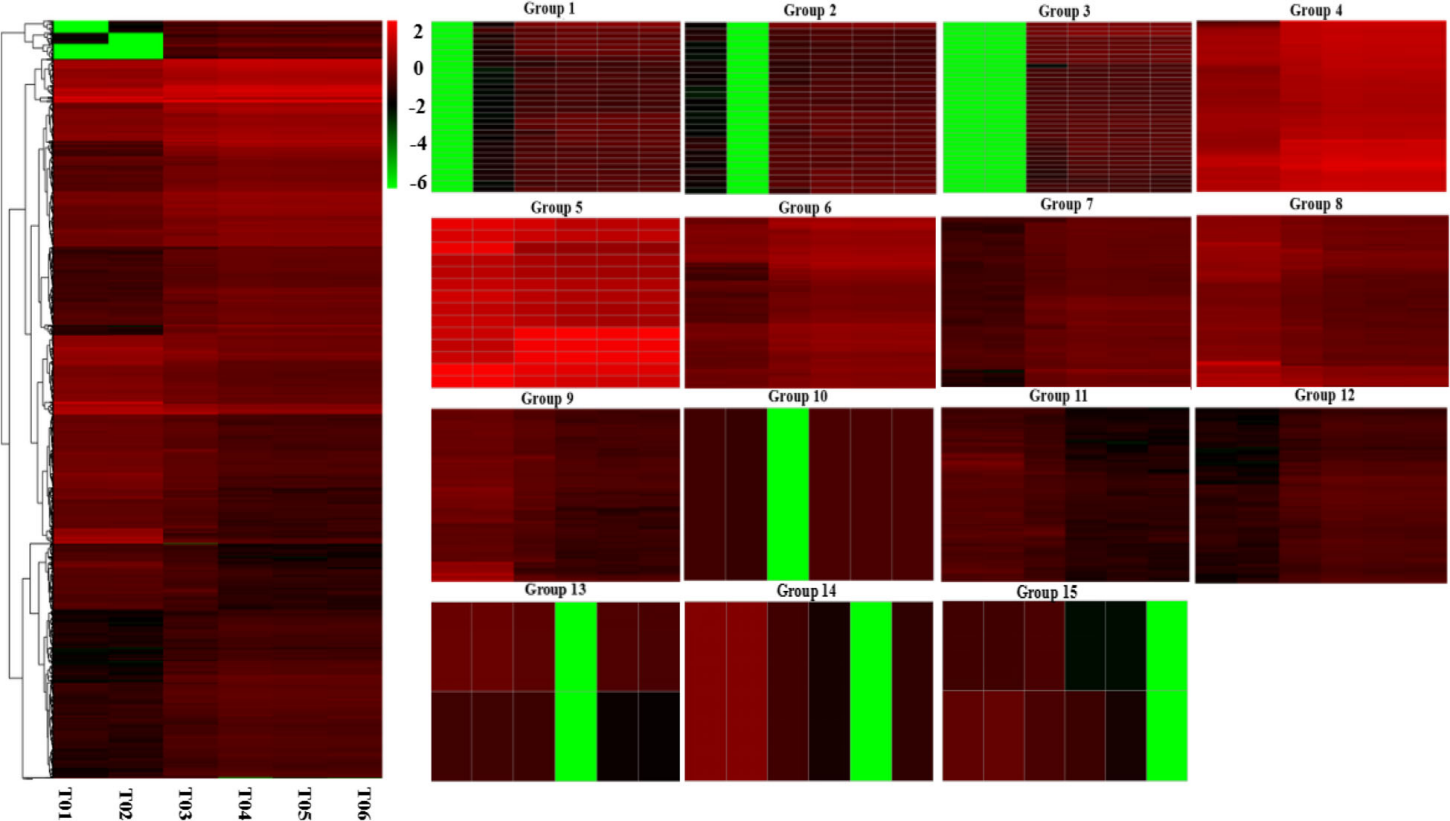
Thermal maps of DEGs and 15 clusters produced for thermographic analysis. Different columns represent different samples and different rows represent different genes. Each small square represents a gene and the color indicates the level of expression; red represents up-regulation and green represents down-regulation

The identified DEGs were analyzed by hierarchical clustering to organize genes with the same or similar expression behaviors to show the different expression patterns of gene sets under different experimental conditions (Fig. 6). In the blueberry kept at 20 °C, most genes were significantly down-regulated; in blueberry kept at 0 °C for 30 days, most of these genes were up-regulated. We clustered these genes into 15 categories according to their functional pathways. These included GABA receptor activity, extracellular-glutamate-gated ion channel activity, and the G-protein coupled receptor signaling pathway. There were 30, 27, 37, 92, 14, 351, 217, 193, 315, 1, 161, 409, 2, 1, 2 genes in each cluster via heatmap analysis, and there were more up-regulated genes in the G4, G5, G6, G7, G8, and G12 clusters, while the down-regulated genes mostly belonged to the G1, G2, G3, G9, G10, G11, G13, G14 and G15 clusters (Fig. 6).

Meantime, the 10 DEGs most significantly changed among treatment groups were selected to assess the important genes and pathways involved in CI (Table 2). Among these DEGs, *c127608* was up-regulated and involved in six pathways and *c120356* was down-regulated and involved in three pathways of the blueberry kept at 0 °C for 30 days.

**Table 2:** Genes significantly up-regulated and down-regulated in 30d-chilled blueberry

Number	Name	Route and name	FC (up)/ (down)
c118333	HSPA1s	ko3040/ko04141/ko04144 protein processing in endoplasmic reticulum	3.428
c76944	Synthase	ko00904 diterpenoid biosynthesis	3.407
c122315	8′-hydroxylas	ko00906 carotenoid biosynthesis	2.582
c120652			2.501
c100356	AGXT2	ko00250/ko00260alanine aspartate and glutamate metabolism	2.485
c122874	MIOX	ko00053/ko00562 ascorbate and aldarate metabolism	2.417
c111137	GH3	ko04075 plant hormone signal transduction	2.404
c59127	INV	ko00052/ko00500 galactose metabolism	2.400
c127608	ALDO	ko00010/ko00030/ko00051/ko00710/ ko01200/ko01230 glycolysis/gluconeogenesis	2.321
c115136	CYP85A2,BR6OX2	ko00905 brassinosteroid biosynthesis	2.305
c120356	HIBCH	ko00280/ko00410/ko00640 valine leucine and isoleucine degradation	−5.716
c89494	CYP82G1	ko00904 diterpenoid biosynthesis	−5.190
c110095	LAR	ko00941 flavonoid biosynthesis	−4.842
c121415	INV	ko00052 galactose metabolism	−4.740
c97853	psbY	ko00195 photosynthesis	−4.640
c111548	CKX	ko00908 zeatin biosynthesis	−4.554
c99806	crtZ	ko00906 carotenoid biosynthesis	−4.201
c129827	G6PDf	ko00480 glutathione metabolism	−3.828
c127697	RP-L5e,RPL5	ko03010 ribsome	−3.807
c111628	RP-L9,MRPL9		−3.787

These genes were selected with an FDR adjusted *P*-value< 0.05

### Functional annotations and classifications

In our research, the N50 was 1348 bp, and there were 21,649 unigenes longer than 1Kb. The assembly integrity was high among the 84,260 unigenes. To predict and analyze the functions of the assembled unigenes, the NR, Swiss-Prot, GO, COG, KOG, Pfam, KEGG, KOG, and eggNOG databases were used as the basic local alignment search tool: 1142 (18.4%) unigenes were annotated in NR; 834 (13.4%) in Swiss-Prot; 685 (11.2%) in GO; 394 (6.4%) unigenes in COG; 922 (14.8%) in Pfam; 413 (6.7%) in KEGG; 638 (10.4%) in KOG; and 1099 (17.7%) in eggNOG.

**Fig. 7:**
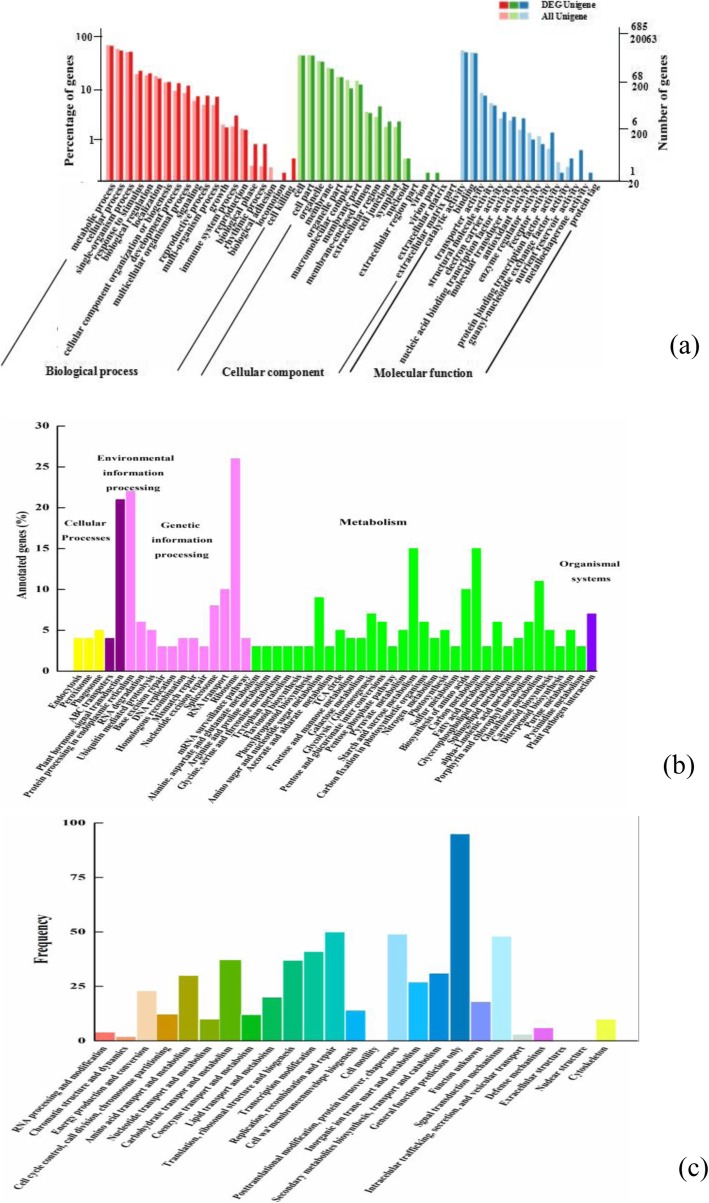
Functional annotations and classifications of unigenes in 30d-chilled blueberry according to (**a**) GO, (**b**) KEGG and (**c**) COG databases

Then the GO enrichment analysis was performed to obtain functional information for the DEGs mentioned above. Significantly enriched GO terms, identified based on corrected *P*-value< 0.05, were found for DEGs involved in biological processes, cellular components, and molecular functions (Fig. 7a). The DEGs found in chilled blueberry were mainly enriched in biological processes, metabolic processes, cellular processes, single organism processes, responses to stimulus, biological regulation, localization, cellular components, developmental processes, and 12 other different biological processes. The DEGs included in the metabolic process, cellular process, and single-organism process categories were not expressed in blueberry that did not pit.

To further identify the functions of DEGs in response to cold stress, the KEGG database was used to classify and characterize the DEGs into corresponding pathways. By comparing the samples, we found that 413 DEGs were enriched into 50 pathways; three of these 50 pathways were related to cellular processes, two pathways belonged to environmental information processing, 12 pathways contained 98 DEGs involved in genetic information processing, 32 pathways included 174 DEGs involved in metabolism, and the remaining pathway was related to organism systems (Fig. 7b). The results of the KEGG analysis showed that, in blueberry under cold stress, 26 DEGs were involved in plant hormone signal transduction pathways, including auxin (Aux), cytokinin (CK), gibberellin (GA), abscisic acid (ABA), ethylene (ET), brassinosteroid (BR), jasmonic acid (JA), and salicylic acid (SA) signaling pathways, each gathering different numbers of DEGs. Moreover, carotenoid biosynthesis, which is involved in the biosynthesis of ABA, comprised five DEGs. These results indicated that not only fatty acid metabolism, but also multiple plant hormone signal transduction pathways were activated under cold stress leading to blueberry fruit pitting. Based on enrichment results, the six most significantly changed pathways in response to cold stress were plant hormone transduction, carotenoid biosynthesis, GSH metabolism, starch and sucrose metabolism, protein processing in endoplasmic reticulum, and chlorophyll metabolism (Table 3).

**Table 3:** The top 6 enriched pathways of DEGs in 30d-chilled blueberry

Pathway	Type	KO_ID	DEGs in pathway	All genes in pathway	*P*-value
Plant hormone transduction	Environmental information	ko04075	21	232	5.23e-05
Carotenoid biosynthesis	Metabolism	ko00906	5	34	0.006
Glutathione metabolism	Metabolism	ko00480	11	134	0.007
Starch and sucrose metabolism	Metabolism	ko00500	15	218	0.009
Protein processing in endoplasmic	Genetic information	ko04141	22	410	0.03
Porphyrin and chlorophyll metabolism	Metabolism	ko00860	6	67	0.03

These were selected with an FDR adjusted *P*-value< 0.05

In addition, 394 DEGs (25classes) were annotated in the COG database (Fig. 7c). The top three COG terms were: general function prediction only (95DEGs); replication, recombination and repair (50 DEGs). Energy production and conversion, amino acid transport and metabolism of carbohydrate transport, inorganic ion transport, and metabolic biosynthesis transport and catabolism were also important.

### Identification and analysis of the DEGs under cold stress

#### Cold-response DEGs involved in membrane lipid and energy metabolism

In this study, six highly differentially expressed pathways of the 129 pathways including membrane lipid metabolism, including glycerolipid metabolism (Ko00561), glycerophospholipid metabolism (Ko00564), ether lipid metabolism (Ko00565), α-linolenic acid metabolism (Ko00592), sphingolipid metabolism (Ko00600), and phosphatidylinositol signaling system (Ko04070) were significantly changed in chilled blueberry. There was one down-regulated DEG in Ko00561; and the Ko00564; Ko00592, Ko00565, Ko00564, Ko00600, and Ko04070 comprised one, one, four, two, and one highly differentially up-regulated expressed structural genes, respectively. Among these six pathways, Ko00564 and Ko00592 were strongly induced in plants under cold stress; *c107765.graphc0*(*Phosphocholine CytidylyltransferaseA*) was down-regulated; the *c100748.graphc0* (*glycerol-3-phosphate dehydrogenase1*), *c131228.graphc1* (*phospholipaseD1/2*), *c125240.graphc0* (*N-myristoyltransferase*), *c126471.graphc0* (*Triacylglycerol lipase4*), and *c130733.graphc0* (*phosphatidylserine synthase2*) were up-regulated in Ko00564, while *c130733.graphc0* was up-regulated in Ko00592.

**Fig. 8:**
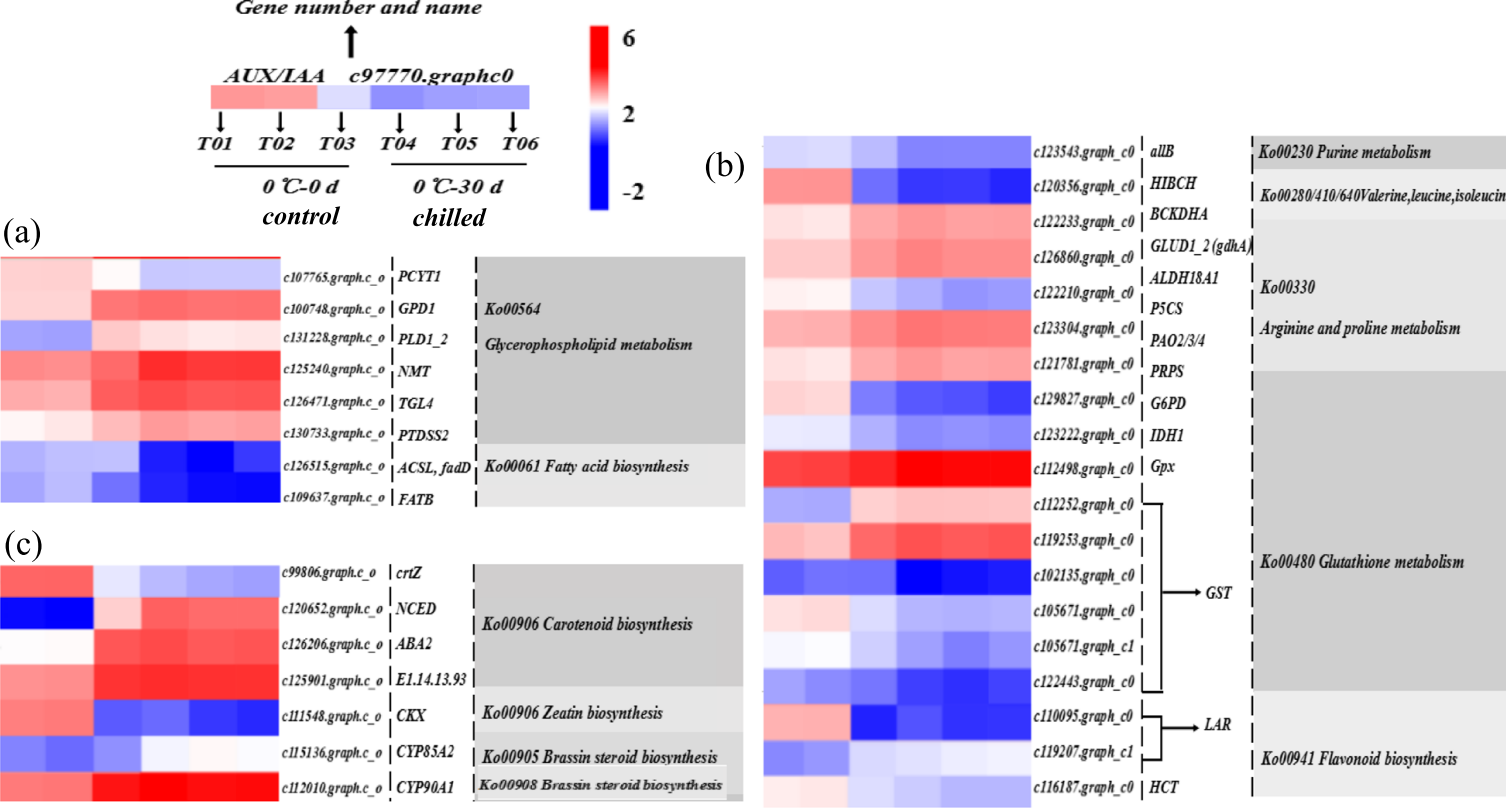
Transcripts involved in (**a**) liquid related pathways, (**b**) proline, glutathione, and flavonoid metabolism, (**c**) brassinosteroid biosynthesis, carotenoid biosynthesis, and zeatin biosynthesis in chilled blueberry

The changes in cell membrane lipid compositions are closely related to fatty acid metabolism under cold stress. Six of the 129 pathways significantly changed in chilled blueberry were related to fatty acid metabolism, which included fatty acid biosynthesis (Ko00061), fatty acid elongation (Ko00062), fatty acid degradation (Ko00071), linoleic acid metabolism (Ko00591), biosynthesis of unsaturated fatty acids (Ko01040), and fatty acid metabolism (Ko01212). There were three, one, and two DEGs down-regulated in Ko00061, Ko00071, and Ko01040, respectively; there were one, one, five, one, and two DEGs up-regulated in Ko00062, Ko00071, Ko00591, Ko01040, and Ko01212, respectively. Genes *c126515.graphc0* (*Fas-associated protein with death domain*), *c109637.graphc0* (*fatty acyl-ACP thioesteraseB*), *c126471.graphc0* (*doxycycline*) were down-regulated in Ko00061 (Fig. 8a).

In the Ko00230 purine metabolism pathway, the DEGs related to energy metabolism affected by cold stress. Gene *c121781.graphc0*, which was up-regulated, participates in the pentose phosphate pathway. This is involved in several biosynthesis pathways of microbial metabolism in diverse environments and in carbon metabolism biosynthesis; c*123543.graphc0* was expressed in chilled blueberry and its expression was lower in this group than control fruits. In the Ko00280 valine, leucine, and isoleucine metabolism pathway, *c122233graphc0* was up-regulated; *3-Hydroxyisobutyryl-CoA Hydrolase* was co-regulated by two genes, *c120356.graph* and *c122037.graphc0*, which were significantly down-regulated (fold-change = − 5.71) and are involved in the beta-alanine metabolic and carbon metabolism pathways.

#### Cold-response genes involved in proline, glutathione, and flavonoid metabolism

Additionally, dehydration stress is often accompanied by osmotic adjustment. Plants need to accumulate or reduce organic or inorganic substances such as proline, glutathione, and flavonoid to maintain cellular moisture holding capacity. According to pathway enrichment analysis, arginine and proline metabolism is activated by cold-induced dehydration*.* In our study, *c126860.graphc0* (*NADP-specific glutamate dehydrogenase*) also participates in alanine, aspartate, glutamate metabolism, D-glutamate metabolism, and carbon metabolism, was up-regulated; c*123304.graphc0* (*polyamine oxidase2/3/4*) was up-regulated during spermidine synthesis and it is also involved in beta-alanine metabolism. In our validation experiments, we found that the relative expression of *c126860.graphc0* in chilled blueberry was significantly up-regulated. The expression of c*123304.graphc0* was also significantly up-regulated in chilled blueberry.

In the present study, *c123222.graphc0* [*Isocitrate dehydrogenase1/2* (*IDH1/2*)] plays a role in intermediary metabolism and energy production, might tightly associate or interact with the pyruvate dehydrogenase complex in the citric acid cycle (TCA cycle). Some genes related to glutathione were also identified. GPX and glutathione transferase (GST) are also important scavengers of ROS that participate in many kinds of environmental stress responses to adverse conditions. Glutathione peroxidase participates in arachidonic acid metabolism and, together with *c112252.graphc0* [*glutathione s-transferase* (*GST*)], *c119253.graphc0* (*GST*), *c102135.graphc0* (*GST*), *c105671.graphc0* (*GST*)*, c105671.graphc1* (*GST*), *c122443.graphc0* (*GST*), *c110117.graphc0,* and *c120425.graphc0* coordinates the GST metabolism of xenobiotics by cytochrome P450. In our validation, *GST* and *IDH1* were down-regulated in chilled blueberry compared to the control blueberry. The expression of *GST1* was significantly higher in chilled blueberry; the expressions of *GST2* and *GST3* were significantly lower in chilled blueberry (Fig. 8b).

The gene *c116187.graphc0* [*Hydroxycinnamoyl-CoA shikimate* (*HCT*)] encodes shikimate O-hydroxycinn amoyltransferase (EC:2.3.1.133), which participates in metabolic pathways and secondary metabolite biosynthesis; the leucoanthocyanidin reductases (LAR) encoded by *c119207.graphc1* and *c110095.graphc0* participate in secondary metabolite biosynthesis. Our qRT-PCR results showed that the expressions of *HCT* and *LAR* (*c110095.graphc0*) were down-regulated and that of *LAR* (*c119207.graphc1*) was up-regulated in chilled blueberry. (Fig. 8b).

#### Cold-response genes involved in hormone biosynthesis and signal transduction

##### Brassinosteroid biosynthesis (Ko00905), carotenoid biosynthesis (Ko00906), and zeatin biosynthesis (Ko00908).

In the zeatin biosynthesis pathway, *c111548.graphc0* (*Cytokinin oxidase/dehydrogenase*) was significantly down-regulated to − 4.5 in chilled blueberry; c*126830.graphc0* (*tRNA isopentenyltransferase1*) was up-regulated during the production of uridine phosphorylase and cis-zeatin glucoside. Genes *c112010.graphc0* [*Cytochrome-P450-90A1-*(*CYP90A1*)] and c*115136.graphc0* [*Cytochrome-P450-85A2-*(*CYP85A2*)] participate in secondary metabolite biosynthesis and metabolic pathways, including BR biosynthesis. These two genes had higher expression in chilled blueberry (Fig. 8c).

In the carotenoid biosynthesis pathway, *c99806.graphc0* (*beta-carotene 3H-hydroxylase*), the precursor of ABA synthesis, was significantly down-regulated, while the synthesis and metabolism of ABA, also included in this pathway, and related genes were significantly up-regulated in chilled blueberry. This finding was in agreement with the fact that stress can increase ABA biosynthesis and accumulation as part of the plant defense response. 9-Cis-epoxycarotenoid dioxygenase [NCED; *c126206.graphc0*) is the most critical rate-limiting enzyme in ABA synthesis and it was significantly up-regulated after storage at 0 °C. Xanthoxin dehydrogenase/ABA2 (*c125901.graphc0*) is another rate-limiting enzyme in the ABA synthesis pathway, and genes *c120652.graphc1* and *c122315.graphc1* co-regulate ABA 8′-hydroxylase. The results of our qRT-PCR showed that the expression of beta-carotene 3-hydroxylase was up-regulated in chilled blueberry. The *ABA2* genes were significantly up-regulated in chilled blueberry. Expression of *Cytochrome P450 707A* (*CYP707A*), which encodes a cytochrome P450 that participates in plant metabolism of terpenoids and polyketides in the ABA metabolic pathway, was significantly up-regulated in chilled blueberry and was 25 times that measured in control blueberry. Thus, cold storage stimulated the expression of genes that encode ABA synthase-related enzymes and are involved in ABA synthesis, but also promoted its ABA metabolism to dihydroxy phaseic acid. Pitting in blueberry after a 30 days’ storage at 0 °C might be affected by these genes; however, the specific mechanism needs further study (Fig. 8c).

##### Plant hormone signal transduction analysis

**Fig. 9:**
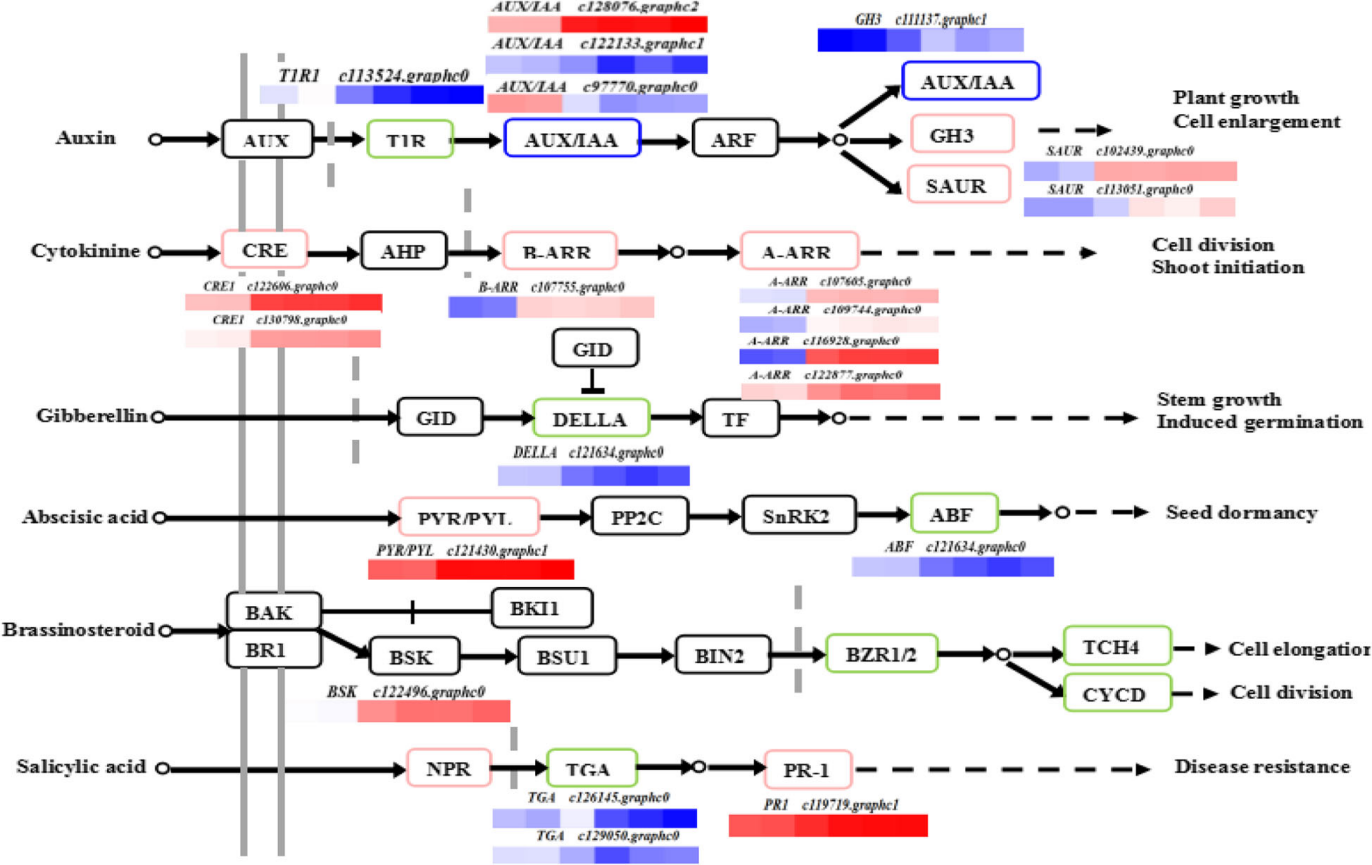
Transcripts involved in hormone related pathways in 30d-chilled blueberry

The results of transcriptome analysis indicated a large number of DEGs were enriched in the plant hormone signal transduction pathways, especially in IAA signal transduction, ABA signal transduction, SA signal transduction and GA3 signal transduction (Fig. 9).

Our results indicated that stress-response pathways interact with auxin regulation through the transcription of the *Aux/IAA*, *IAA5*, *IAA6,* and *IAA19* are required for stress tolerance; one gene was down-regulated and two genes were up-regulated in the auxin signaling pathway in chilled blueberries. Gene *c113524.graphc0* encodes transport inhibitor response1 was down-regulated in chilled blueberry. Genes *c128076.graphc0* (*IAA*), *c122133.graphc1* (*IAA*), and *c97770graphc0* coordinate the regulation of IAA, an auxin-responsive protein that participates in plant hormone signal transduction; c*111137.graphc1*, a member of the *GH3* gene family, showed higher expression in chilled blueberry. Genes c*102439.graphc0* and *c113051.graphc0* belong to the small auxin-upregulated RNA gene family, and are regulated by auxin and environmental factors; these genes were similarly expressed with *IAA*, which was significantly up-regulated in chilled blueberry. Pyrabactin resistance 1-like (PYL) is an ABA receptor, *PYL1*, *PYL4*, *PYL5*, *PYL6*, *PYL7*, *PYL10*, *PYL11*, *PYL13*, and *PYL15* have been isolated and identified from *Arabidopsis thaliana*. In the present study, the expression of *c121430.graphc0,* an ABA receptor of the PYR/PYL family involved in mitogen-activated protein kinase signaling, was significantly higher in chilled blueberry fruits. The upregulation of both genes indicated that ABA biosynthesis and catabolism were activated by low temperature. The *ABA response element binding factor* gene (*c112990.graphc0*) was down-regulated in our transcriptome results, while the *brassinosteroid- signaling kinase* (*c122496.graphc0*) was up-regulated in chilled blueberry fruits which was eight times higher than that in control blueberry.

In the SA pathway, gene expression of the plant-pathogen interaction genes *c129050.graphc0* (*Pathogenesis related protein1*) and *c126145.graphc0* (*TGA*) were down-regulated in chilled blueberries. The expression pattern of both genes was similar. Moreover, the treatment of horticultural crops and fruits with GA3 is known to alleviate CI symptoms after treatments at 0 °C [29]. In the GA3 pathway, the expression of *DELLA*, a negative regulator of GA3 signaling, was up-regulated after 30 days’ storage at 0 °C. Three genes were up-regulated in the CK pathway, a CK receptor and the 2-component response regulators ARR-B or ARR-A. The expression levels of *c107755.graphc0* (*ARR-B*) and *c107605.graphc0* (*ARR-A)* in chilled blueberries were significantly up-regulated, and 18 and 59 times that in control blueberries, respectively. Therefore, genes in hormone signal transduction pathways were significantly affected by cold storage at 0 °C, especially those involved in CK and Aux regulation and metabolism.

### TFs in response to cold stress

**Fig. 10:**
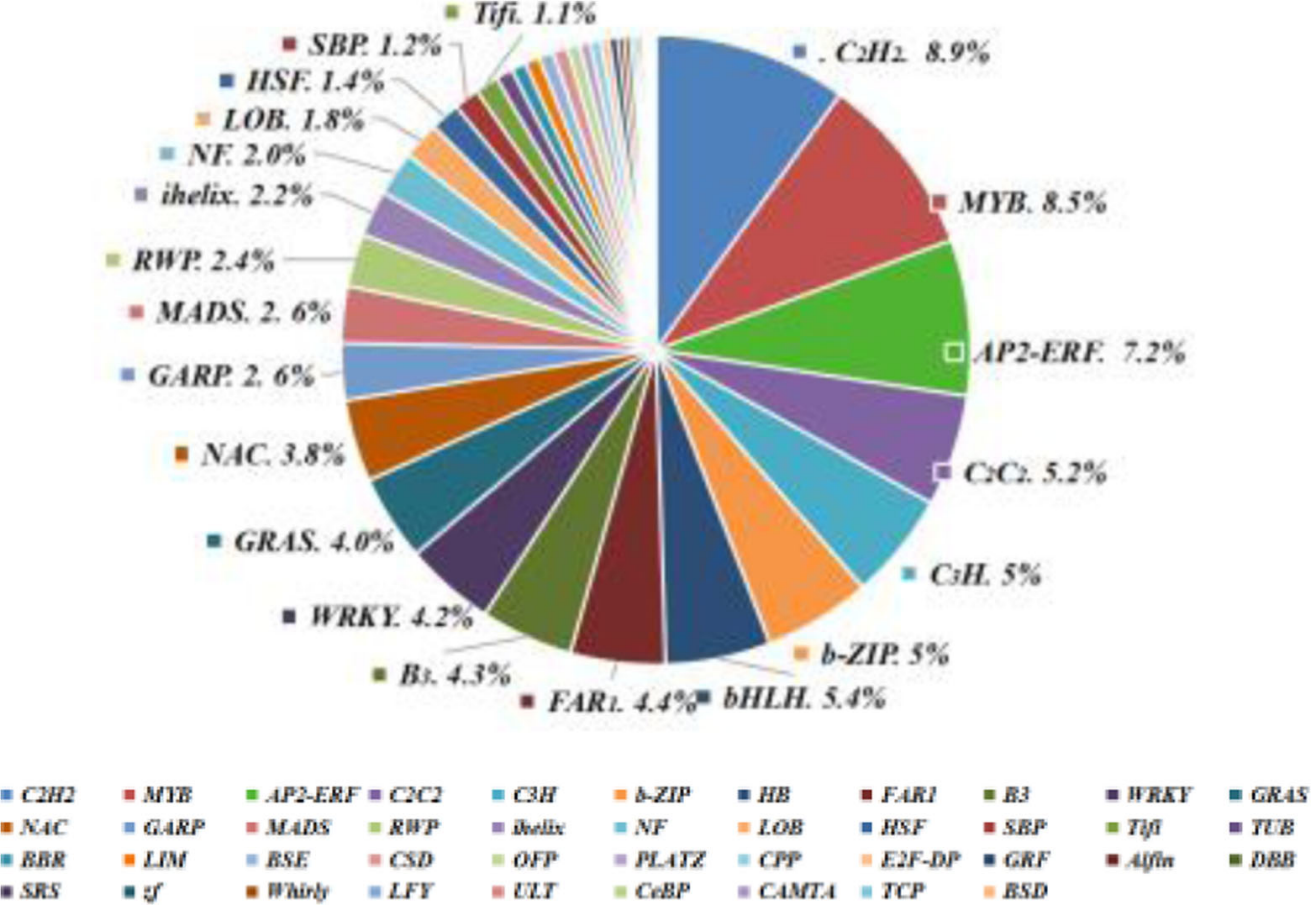
The numbers and classification of differentially expressed TFs in chilled blueberries. Categories of transcription factors less than 1% of the total are not marked on the pie chart

The different gene expression patterns across the 30d-chilled blueberry indicated that multiple structural genes have contributed to blueberry fruit pitting. In the present study, we screened our assembled transcripts and predicted a total of 1023 TFs from 45 families and identified 738 protein kinase, and 327 transcriptional regulators (TRs); the expressions of most of them in chilled blueberry fruits were changed. The 1023 TFs comprised 42 categories of TFs including 92 C2H2, 87 MYB 68, 74 Ap2/erf-erf, 56 bHLH, 53 C2C2, 51 bZIP, 51 C3H, 45 FAR1, 43WRKY, 39 NAC (Fig. 10).

### Validation of the RNA-Seq results by qRT-PCR

**Fig. 11:**
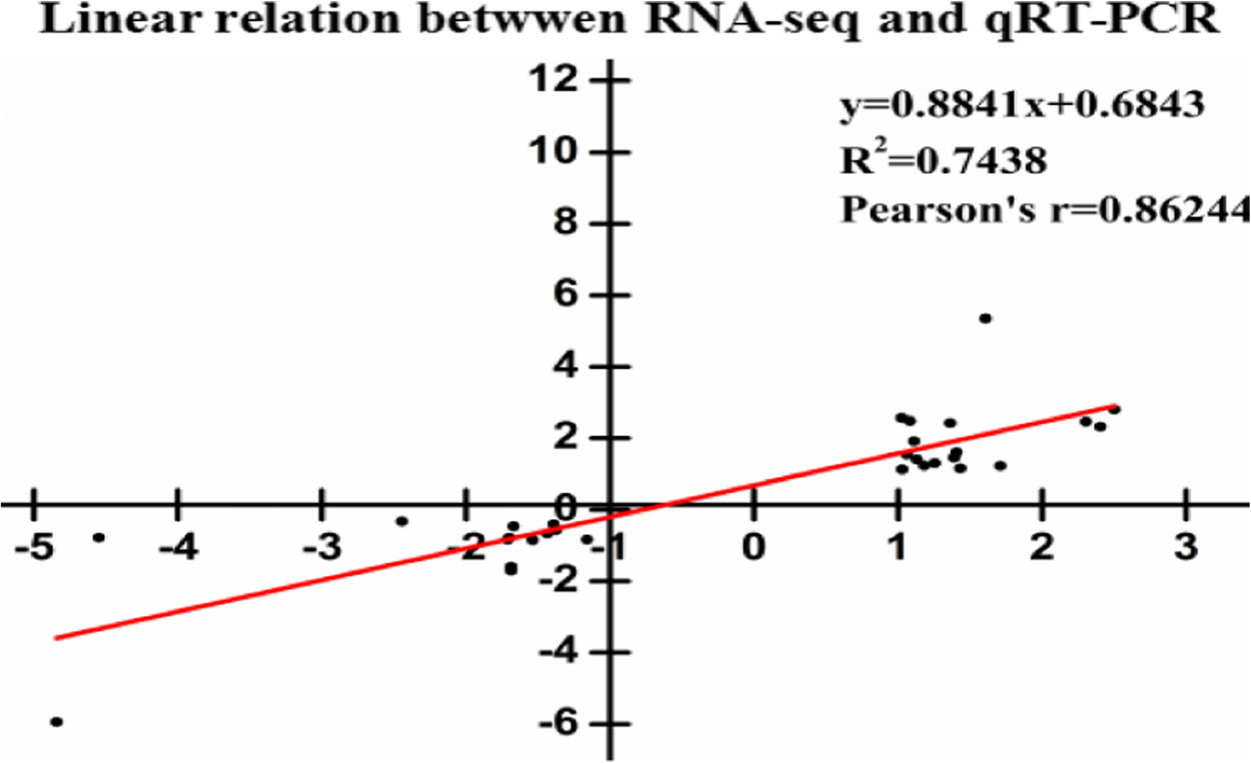
The correlation analysis between qRT-PCR of RNA-Seq results of 40 randomly genes

**Fig. 12:**
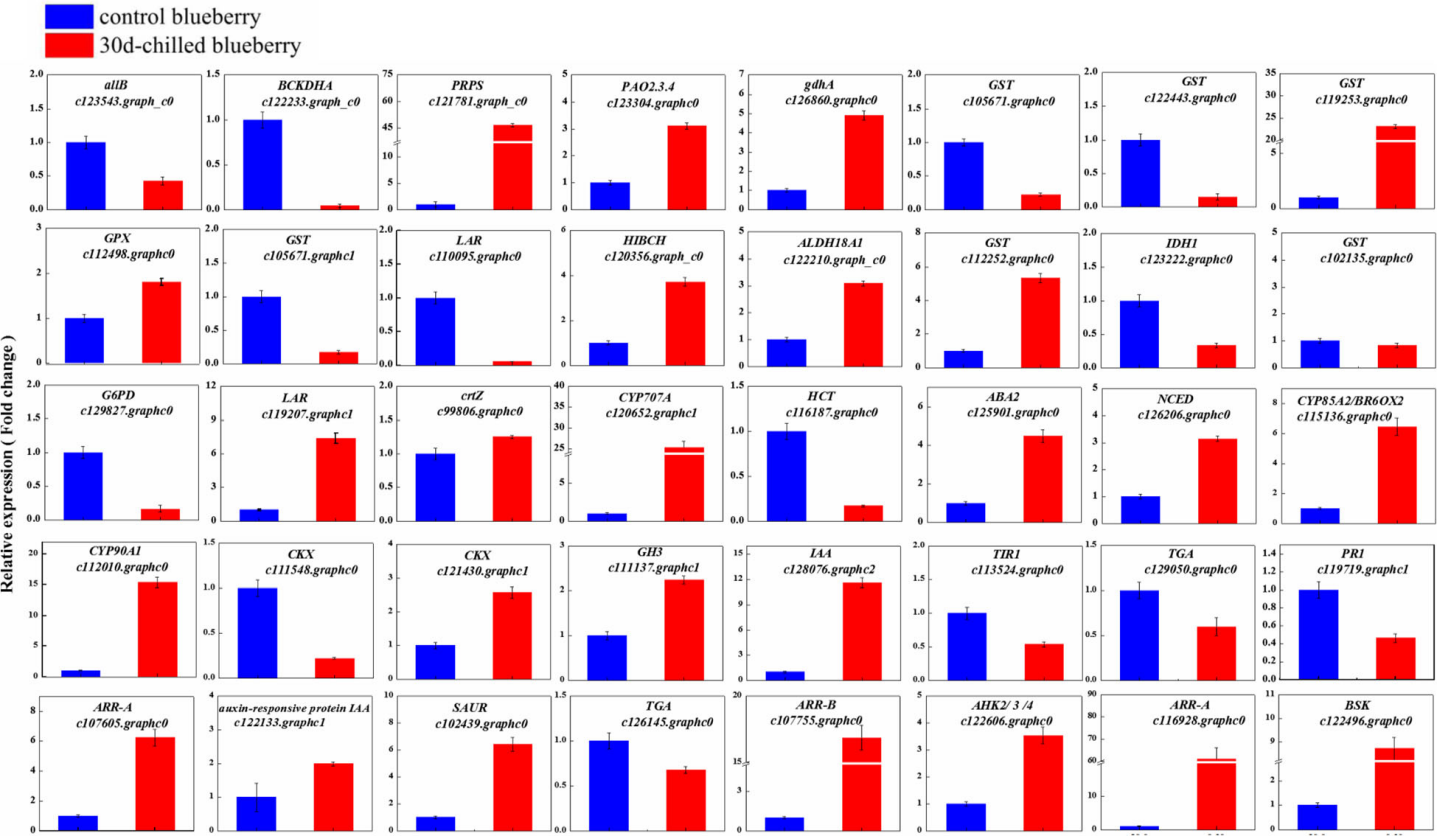
qRT-PCR verification of expression pattern obtained by RNA sequencing. The relative transcription level was calculated according to the 2^−ΔΔCT^ method with actin reference genes as control. Each value is the mean ± SD; *P* < 0.05 (n = 3, three biological replicates, each with three technical replicates)

To ensure the reliability of the RNA-Seq data, the expression patterns of 40 random DEGs were evaluated by qRT-PCR. (Figs. 11 and 12). The genes represented various functional categories or pathways, including liquid related, defense systems, flavonoid metabolism, brassinosteroid biosynthesis, carotenoid biosynthesis, zeatin biosynthesis and hormone signal transduction pathways. The linear regression showed that the results from RNA-Seq and qRT-PCR were highly relevant (Pearson’s *r* = 0.8624), despite the difference in the absolute FC between the two methods. The results showed the expression patterns derived by both methods were consistent, confirming the reliability of the transcriptome results. These consistent expression patterns further confirmed the transcriptome data reported in this study constitutes a valuable supplement to the available blueberry genomic and transcriptome information.

## Discussion

Although LT storage is the major method to prevent most fruits and vegetables from decaying, it may cause CI symptoms, such as the surface pitting and dark watery patches in harvested cucumber [16], peel pitting in banana [8], and less aroma in pear [13]. In our present study, the blueberry were stored at low temperature (0 ± 0.5 °C) for 15, 30, 45, and 60 days. This method can preserve the fruit quality and extend their postharvest life. However, pedicle pitting developed due to LT storage and the cold stress; the longer the blueberries are stored, the more serious the pitting is. And the 0 °C-30 days storage was the critical point of pitting. With the occurance of pitting, the phenotype of fruit changed accompanied with the wrinkled cell membrane and the destroyed structure [30]. Meantime, the physiology indices of EL, MDA, proline content, and GSH content, which were considered to reflect physiological state of plant exposure to cold stress [31], were also determined in our study. And the significant increase in EL, content of MDA and proline, and the decrease in GSH content in pitted blueberry indicated that membrane lipid metabolism plays an important role in cold stress response. However, this is only the changes in physiological.

Although cold stress responses and physiological changes have been extensively studied [6, 7, 9, 10], the its molecular and genetic mechanisms remain poorly characterized in blueberry. In order to explore the direct cause of fruit pitting and the gene regulation net under cold stress at the molecular level, we performed deep transcriptome sequencing of the chilled blueberry and control blueberry samples. According to the RNA-Seq data, the three independent biological replicates were highly consistent (0.9462 < R^2^ < 0.9998), which indicated that the RNA-Seq data was reliable. The transcriptome sequences were assembled into 35,060 transcripts, of which 73.7% were annotated. The annotations provide a valuable resource for investigating specific processes, functions, and pathways during blueberry research. For further verification, we used qRT-PCR, and the expression patterns of 40 significantly changed DEGs corresponded (Pearson’s r = 0.8624) in both methods. Under cold stress, numerous DEGs participating in diverse cell, molecular, and biological pathways were identified as putatively involved in pitting. We mainly focused on the genes involved in lipid metabolism, energy metabolism, antioxidant defense system, hormone signaling networks and TFs based on enrichment results (Tables. 4, 5, 6 and Figs. 8, 9 and S1). These results demonstrate that our transcriptome database is a rich resource for mining cold stress-responsive genes. These transcriptome analysis results provide key points to explain the mechanism underlying the metabolic changes in pitted blueberry in response to cold stress at the molecular level.

**Table 4:** Genes involved in lipid related pathways in in response to low temperature

Name	Gene ID	FC	Definition	Name	Gene ID	FC	Definition
PCYT1	c107765.graphc0	−1.3	choline-phosphate	TGL4	c126471.graphc0	1.12	triacylglycerol lipase SDP1-like
GPD1	c100748.graphc0	1.14	[NAD(+)]	PTDS2	c130733.graphc0	1.17	Phosphatidyl serine synthase
PLD1/2	c131228.graphc1	1.33	Phospholipase-D-p1	fadD	c109637.graphc0	2.22	palmitoyl-acyl carrier protein
NMT	c125240.graphc0	1.45	N-methyltransferase1	FATB	c126515.graphc0	−2.5	acyl-CoA synthetase1

These genes were selected with an FDR adjusted P-value< 0.05

**Table 5:** Genes involved in proline, glutathione, and flavonoid in response to low temperature

Name	Gene ID	FC	Definition	Name	Gene ID	FC	Definition
allB	c123543.graphc0	−1.4	allantoinase	IDH1	c123222.graphc0	−1.44	isocitrate dehydrogenase
HIBCH	c120356.graphc0	−5.7	3-hydroxyisobutyryl-CoA hydrolase	gpx	c112498.graphc0	1.26	glutathione peroxidase
BCKDHA	c122233.graphc0	1.14	2-oxoisovalerate dehydrogenase E1	GST	c112252.graphc0	1.61	glutathione S-transferase
gdhA	c126860.graphc0	1.12	NAD(P)+		c119253.graphc0	1.50	
P5CS	c122210.graphc0	−1.7	delta-1-pyrroline-5-carboxylate		c102135.graphc0	−1.68	
PAO2/3/4	c123304.graphc0	1.03	polyamine oxidase		c105671.graphc0	−1.71	
PRPS	c121781.graphc0	1.41	ribose-phosphate pyrophosphokinase		c105671.graphc1	−1.72	
G6PD	c129827.graphc0	−3.81	glucose-6-phosphate 1-dehydrogenase		c122443.graphc0	−1.54	

These genes were selected with an FDR adjusted *P*-value< 0.05

**Table 6:** Genes involved in plant hormone signal transduction in response to low temperature

Name	Gene ID	FC	Definition	Name	Gene ID	FC	Definition
CYP85A2	c115136.graphc0	2.31	brassinosteroid-6-oxidase 2	GH3	c111137.graphc1	2.41	auxin responsive family
CYP90A1	c112010.graphc0	1.32	cytochrome P450 family 90	TIR1	c113524.graphc0	−1.7	transport inhibitor response 1
crtZ	c99806.graphc0	−4.2	beta-carotene3-hydroxylase	SAUR	c102439.graphc0	1.41	SAUR family protein
NCED	c126206.graphc0	1.43	9-cis-epoxycarotenoid	AHK2/3/4	c122606.graphc0	1.38	cytokinin receptor
ABA2	c125901.graphc0	1.12	Xanthoxin dehydrogenase	ARR-B	c107755.graphc0	1.43	ARR-B family
E1.14.13.93	c120652.graphc1	2.51	(+)-abscisic acid 8′-hydroxylase	ARR-A	c107605.graphc0	1.18	ARR-A family
CKX	c111548.graphc0	−4.5	cytokinin dehydrogenase		c116928.graphc0	2.17	
LAR	c110095.graphc0	1.39	leucoanthocyanidin reductase	PYL	c121430.graphc1	1.02	ABA receptor PYR/PYL
	c119207.graphc1	−4.8		BSK	c122496.graphc0	1.71	BR-signaling kinase
HCT	c116187.graphc0	−1.1	O-hydroxycinnamoyltransferase		c129050.graphc0	−1.4	
IAA	c128076.graphc2	1.13	auxin-responsive protein IAA	TGA	c126145.graphc0	−2.45	transcription factor TGA
	c122133.graphc1	−1.71		PR1	c119719.graphc1	1.14	pathogenesis-protein 1

These genes were selected with an FDR adjusted *P*-value< 0.05

### Lipid metabolism and energy metabolism under cold stress

When the environmental temperature decreases, physical phase and cell arrangement change first; then membrane permeability, lipids’ composition and content begin to degrade. Electrolyte leakage is an important indicator of cell membrane permeability and is the most frequently used method to evaluate plant tissue injury under severe stress conditions. The substantial increase in EL is commonly caused by changes in membrane structure and lipid composition and considered as an index reflecting cell membrane function and integrity [32, 33]. In our present study, the severity of pitting in blueberries was related to relatively higher EL. In addition to electrical leakage, the lipid peroxidation are also evaluated in studies of fruit mechanisms under cold stress [34]. Since MDA content is a direct measure of lipid peroxidation, it can be evaluated to assess the degree of cell damage [35]. In the present study, we observed a significant increase in MDA content during cold storage, especially during the shelf life after 0 °C storage. These clearly indicated that blueberry membrane lipid peroxidation occurs during LT storage and it is intensified during shelf life after 0 °C storage.

Meantime, the accumulation of proline is also related to the cold tolerance in most plant [36]. And the proline has been confirmed to accumulate when plants are exposed to salt or low temperature stress [37]. In our present study, the proline content of blueberries after 30 days’ cold storage was significantly higher than that of other blueberries during the 8-days shelf life; additionally, the proline content of blueberry fruits after 30 days’ cold storage was more than 2 times higher than the blueberries in the other group. Obviously, the increase of proline provided strong evidence that LT storage caused cold stress and blueberries responded to cold stress. In the meantime, we also observed that the GSH in the cold storage group had a downward trend while that in the other group had an upward trend. In conclusion, membrane lipid metabolism plays an important role in blueberry response to cold stress and may be crucially related to the tolerance of fruit to cold stress. This was consistent with some studies in other fruits. Li et al. (2012) [38] confirmed relative EL had a remarkable increase in the mango fruit stored at 5 °C. Kong et al. (2017) [4] also indicated that LT (4 °C) caused serious membrane damage in peppers and the MGDG, PC, PE and PA changed in response to cold. Wang et al. (2019) [30] also identified the blueberries had a higher level of DGDG after LT storage. Additionally, changes of fatty acids were also involved in membrane lipid metabolism. And the change of the lipid not only occurs to the fruits and vegetables in the post-harvest low-temperature storage process, but also occur to the fruits and vegetables or the plants during the cold acclimation [39].

Additionally, genetic and molecular evidence shows that pitting is a complex phenomenon involving the alteration of metabolism with synthesis of specific metabolites, lipids, energy and other pathways. And the changes in gene expression underlie some of the biochemical and physiological changes that occur during cold storage. In the present study, the genes regulating membrane lipid components and fatty acids also significantly changed under cold stress. Six highly differentially expressed pathways of membrane lipid metabolism, including glycerolipid metabolism (Ko00561), glycerophospholipid metabolism (Ko00564), ether lipid metabolism (Ko00565), α-linolenic acid metabolism (Ko00592), sphingolipid metabolism (Ko00600), and phosphatidylinositol signaling system (Ko04070) were significantly changed in chilled blueberries. Additionally, six highly differentially expressed pathways related to fatty acid metabolism including fatty acid biosynthesis (Ko00061), fatty acid elongation (Ko00062), fatty acid degradation (Ko00071), linoleic acid metabolism (Ko00591), biosynthesis of unsaturated fatty acids (Ko01040), and fatty acid metabolism (Ko01212) were also significantly changed in chilled blueberries. Among these genes, the *fadD*, *c109637.graph_c0* encoding palmitoyl-acyl carrier protein was the most obvious up-regulated gene, its expression was 2.2 times higher than that in the control group; the *FATB*, *c126515.graph_c0* encoding acyl-CoA synthetase 1 was the most obvious down-regulated gene, its expression was 2.5 times lower than that the control group. These results suggested the pathways related to membrane lipid had a strong response to cold stress, which was consistent withe the results in loquat [21] and these two genes may be the most ideal genes to further study the regulation of membrane lipid metabolism response to fruit pitting. The study of Die and Rowland. 2014 [40] also confirmed the pathways associated with lipid metabolism, especially regarding enzymes involved in fatty acid elongation, glycerophospholipid metabolism and -linolenic acid metabolism changed significantly in response to cold stress. Moreover, Die and Rowland. 2014 [40] identified the genes encoding phospholipaseC, histidine kinase, 5 lipid-transfer protein (LTP) genes and 2 fatty acid desaturases with potential roles in altering the composition of proteins and lipids.

In fact, the occurrence of CI is not just the results in detrimental changes in membrane structure but also related to the changes of energy status [2, 41]. Published data on other fruits also suggest a close association between membrane integrity regulation is cellular energy status. It has been suggested that the damage of cell membranes was associated with lack of energy status, and ATP played important roles in synthesis of fatty acid and repair of membranes [42]. Recently, many evidences have shown that development of CI in postharvest fruit is partly attributed to limited availability of energy or low energy production, whereas acquaintance of higher levels of ATP and energy charge alleviate CI [16, 43]. Moreover, energy metabolism is important to sustain plant life and plant resistance to environmental stresses [14, 44, 45]. In the present study, there were numerous down-regulated DEGs involved in energy metabolism, which may suggest that the available energy status and the stable enzymatic system in blueberry collectively contribute to improve chilling tolerance by alleviating pitting and maintaining the quality of blueberry fruits during storage at 0 °C [1]. Energy metabolism is linked to adenosine triphosphate (ATP) production and it is the major determinant of cell function and viability, as indicated by plants with higher energy and better maintenance of biological membrane structure being less susceptible to CI [46, 47]. Conversely, long-term storage at 0 °C could result in the decrease of ATP levels in postharvest fruit, causing a disruption in their energy metabolism [38]. In particular, it has been shown that ATP and energy content in papaya decreased after fruit storage at 0 °C, indicating that CI has a close relationship with energy metabolism [45]. Consistent with these findings, our results suggest that energy metabolism might be a main reason underlying the pitting symptoms and therefore exogenous ATP treatment might reduce pitting of blueberry fruits. And it has been proved that the high ATP level can be maintained by increasing the D-pyrroline-5-carboxylatesynthetase (P5CS) activity and then reduce the CI [44]. And suppressing reactive oxygen species [16] modulating proline accumulation [48] may can also enhance the chilling tolerance.

### Hormone signals under cold stress

Previous studies have demonstrated that several plant hormones are involved in modulating response and adaptation to a changing environment in plants [49]. As secondary signals, they can initiate a series of signal events (cascade reactions) that ultimately induce stress responsive genes. They may highly correlated with stress responses under LT after harvest and the changes in plant hormone contents can also affect fruit quality. BRs can confer resistance of plants to various abiotic and biotic stresses [50]. Mango fruits treated with Br had higher cold stress tolerance through the regulation of lipids [44]. And exogenous BL can markedly decreased CI incidence of mango fruit. In addition, other plant hormones, including ABA, GA3, JA and ethylene [51, 52], have been also implicated to modulate abiotic and biotic stresses. MeJA treatment can effectively inhibit the CI in postharvest loquat fruit [53, 54] and exhibit higher tolerance to cold stress in postharvest peach fruit by inducing enzyme activities related to energy metabolism and maintaining high levels of ATP and energy charge [55]. Recent studies revealed that exogenous application of SA was able to significantly remit chilling symptoms in harvested loquat fruit [56] and mango fruit. And treatment with ETH or BR, at an effective concentration, could also alleviate CI of tomato fruit [57, 58]. And the role of ABA in the stress-resistant process of the plant is also essential. The content of ABA increases during plant defense responses and this phytohormone plays multiple roles in plant stress responses to drought and cold [59]. ABA is also involved in regulation of cold acclimation. The ABA-insensitive 5 (ABI5) protein was one of the 13 functionally and structurally-related bZIP proteins found to play crucial roles in mediating changes in stress responses [60]. Thus, plant hormones have an important role in low temperature tolerance in postharvest fruit.

In the present study, we found numerous plant-hormone-related genes were responsive to CI. The numbers and expression patterns of these genes are shown in (Fig. 9); in particular, genes involved in BR, CK, Aux, and SA pathways were up-regulated while those involved in ET and methyl jasmonates pathways were not affected by CI. But it may be not consistent with Ding et al., 2015 [51], they revealed that, during cold storage, endogenous levels of GA3 and the expression level of the key GA metabolic genes were lower in chilled fruit compared to that of fruit stored at room temperature. Perhaps because of the different species, the trend of hormone content change under low temperature stress is also different. Additionally, genes involved in Aux and SA regulation were similarly affected CI, probably because the expression of receptor genes decreased, but the expression of PR1 and GH3 increased. Overall, ABA, GA3, BRs, and CK played important roles in pitting caused by cold storage by regulating the expression of numerous downstream genes in blueberries. These results indicate that hormone signaling pathways may play unique functions under cold stress conditions, and may be forming a complex antioxidant defense system in blueberry. However, whether and how ABA, GA3, BRs, or CK mediated signaling participates in cold stress responses in blueberry need to be further determined.

### TFs involved in blueberry pitting responses to cold stress

In many biological processes, defense responses also require the regulation of specific TFs, which comprise one of the complex regulatory networks in plants [14]. TFs may play essential roles in stress responses by regulating their target genes through specific binding to cis-acting elements in their promoters. Although the TFs families play diverse roles in plant developmental processes and environmental responses, most of them have been reported to be linked to cold stress resistance in plants. For example, six *BrbZIP* genes have been identified as putative key factors in cold stress response [23]. NAC genes are also reported to be induced by cold in *Arabidopsis* [61], *chrysanthemum* [62], rice [63] and *sugarcane* [64]. Additionally, the NAC TFs can also interact with CBF1 to regulate cold tolerance in apples [65] and bananas [66]. The bHLH family, the second largest family [67], can also increase cold tolerance in plants by regulating the expression of ROS clearance-related and stress-responsive genes. Meanwhile, the first WRKY transcription factor was found in sweet potato under cold stress [68]; in cucumber, CsWRKY46 confers cold tolerance and positively regulates ABA-dependent cold signaling pathways [69].

Some less common TFs like C2H2-zinc finger proteins are also essential in plant stress responses, although their transcriptional regulatory mechanisms remain largely unclear [70]. Overall, our results revealed that the 1023 TFs comprised 42 categories of TFs including 92 C2H2, 87 MYB 68, 74 Ap2/erf-erf, 56 bHLH, 53 C2C2, 51 bZIP, 51 C3H, 45 FAR1, 43WRKY, 39 NAC were changed in the pitting process; this was a little similarity with Die and Rowland (2014) [40]; they found the seven most highly represented TF families in response to cold stress were WRKY, ARF, C3H, AP2/ERF, bHLH, C2H2, and NAC. Based on our current results, the bHLH family was significantly up-regulated in chilled blueberries, but Ap2, ZIP, and WRKY families were significantly down-regulated under this condition. In addition, Plant hormones and transcription factors may also interact during cold stress. For instance, NAC TFs have been demonstrated to regulate the expression of ABA-related genes during abiotic stress responses [71]. And ethylene was shown to negatively regulate cold signaling at least partially through the direct transcriptional control of CBFs [52]. Since our current research focus on further confirming whether the C2H2, MYB, and NAC TFs play an important role in the response to LT, and how they regulate the expression of downstream genes in pitted blueberry.

## Conclusion

Overall, the harvest longterm cold storage leads serious CI in blueberries, which substantially decreases the quality, storability and consumer acceptance. Our results identified the occurrence of pitting, and proved 30 days’ cold storage is the critical point of pitting. We found higher MDA content, proline content, EL and lower GSH content in 30d-chilled berries. Additionally, a comprehensive transcriptome profile of blueberry under cold stress were explored by RNA-seq this time in order to characterize the CI mechanism at the molecular level. We identified and summarized the genes in response to cold stress as follows: (1) transcription factors; (2) membrane lipid and energy metabolism; (3) defensive; and (4) hormone biosynthesis and signal transduction, including the genes involved in membrane lipids, proline, glutathione, flavonoids, brassinosteroid, carotenoid, and zeatin biosynthesis pathway. These DEGs and the biological processes related to the stress responses, lipid and hormone metabolic processes almost all accumulated and up-regulated in the CI process. And may play a crucial role in the response to cold stress in blueberry. These results provides novel insights into a series of molecular mechanisms underlying physiological metabolism and defense. Further, the enormous amount of blueberry transcriptome data generated here will serve as the foundation in finding available effective way in alleviating CI and also help to perfect the lack of the genetic information of non-model plant species. In future work, we aim to focus on the function verification of special structural genes fadD (c109637.graph_c0) related to membrane lipid and fatty acid metabolism and 8′-hydroxylas (c122315.graph_c0) related to plant hormone and explore how they work in chilling process.

## Methods

### Plant materials and temperature treatments

‘Duke’ blueberry (*Vaccinium corymbosum L.*) fruits were harvested at commercial maturity from an blueberry base in Shenyang (N41°39′55.82″, E123°05′12.46″), Liaoning Province, China, from April to July in 2016 to 2018. The blueberries were manually picked to prevent any mechanical damage. After 2 h pre-cooling at 20 °C, blueberry fruits without physical injury, disease, or rot were enclosed in plastic boxes; then stored at different storage temperatures (20 °C and 0 °C). Each box contained 125 g blueberry fruits and the relative humidity was kept at 85%. The fleshy tissues were collected, quickly frozen in liquid nitrogen, and stored at − 80 °C [72]. The blueberry samples non-pitted (T01, T02, and T03) were defined as control blueberry and the pitted blueberry stored at 0 °C for 30 days (T04, T05, and T06) were defined as 30d-chilled blueberry. They were collected for RNA-Seq and the RNA-Seq was did by Biomarker Technologies (Beijing, China). During this period, we ground 20 different blueberry fruits of each samples (T01–06), and in each samples (T01–06), we also perform three technical replicates.

### Evaluation of pitting rate, decay rate, electrolyte leakage, MDA and proline

Pitting and decay rate were calculated for fruits at 0, 2, 4, 6, and 8 days shelf-life, with or without previous storage at 0 °C, using the following equations:

1 $$ \mathrm{Pitting}\ \mathrm{rate}\ \left(\%\right)=\mathrm{An}/\mathrm{Am}\times 100 $$

2 $$ \mathrm{Decay}\ \mathrm{rate}\ \left(\%\right)=\mathrm{Ai}/\mathrm{Am}\times 100 $$

In these equations, An is the number of blueberry fruits with pitting, Ai is the number of blueberry fruits cankered, juicy, or mildew, and Am is the total number of blueberry fruits. Three independent measurements were performed at each time point for 150 fruits per replicate sample.

The cell membrane permeability determined by electrical conductivity meter according to the method described in Mao et al. (2007) [73]. MDA content was determined according to the method described in Zhao et al. (2005) [74]. The proline (Pro) content and reduced glutathione (GSH) content were determined according to the method described in Zhao et al. (2009) [75].

### Total RNA isolation, cDNA library construction, and sequencing

Total RNAs from all samples were isolated using the OminiPlant RNA kit (CWBio, Beijing, China). The integrity was monitored on 1% agarose gels and the purity was checked using the NanoDrop spectrophotometer (Thermo Fisher Scientific, Inc.) according to Niu et al. (2017) [76]. Sequencing libraries were generated using NEBNext®Ultra™ RNA Library Prep Kit (Illumina®NEB, USA) and index codes were added to attribute sequences to each sample. The library quality was assessed on the Agilent Bioanalyzer 2100 system. Finally, the cDNA libraries were sequenced on the Illumina® HiSeq 2000 platform (Illumina Inc.) and paired-end reads were generated at Beijing Biomarker Technology Company (Beijing, China) [77]. All the downstream analyses were based on clean data with high quality. And all the sequencing data generated in this study have been deposited in the NCBI with the link of http://www.ncbi.nlm.nih.gov/bioproject/578101, under the accession number SAMN13049031, the Temporary Submission ID SUB643610 and the BioProject ID PRJNA578101.

### Functional annotation and differential expression analysis of genes

Because of the absence of a accurate reference-grade genome for blueberry, a transcriptome was de novo assembled from this RNAseq data and other publicly available transcriptome data from blueberry downloaded from the National Center for Biotechnology Information (NCBI). And the non-redundant unigenes were annotated based on NCBI non-redundant (NR); Protein family (Pfam); Swiss-Prot, Clusters of Orthologous Groups (KOG/COG/eggNOG); Kyoto Encyclopedia of Genes and Genomes (KEGG) and Gene Ontology (GO) databases according to Liu et al. (2015) [77].

DESeq R package (1.10.1) was used to identify the DEGs between control and chilled groups with the standard *P*-value< 0.05 and |log2 (fold change)| ≥ 1. The GO enrichment and KEGG analysis of the DEGs was performed to identify significantly enriched biological pathways of DEGs, with an FDR adjusted *P*-value< 0.05.

### Validation of the RNA-Seq data and expression analysis by qRT-PCR

Quantitative real-time PCR was performed to validate the expression patterns obtained from the RNA-Seq data. Gene specific primers were designed in Primer 5.0 and synthesized by Genewiz Biotechnology Synthesis Lab (Jiangsu, China) (Table S1 Primers used in qRT-PCR). The program and reaction conditions for the 2 × ULtraSYBR Mixture (Low ROX) Kit (ComWin Biotech, Beijing, China) using QuantStudio 6 Flex (Life Technologies) were as described in the manufacturer protocols. Template cDNA was diluted five-fold, and 2 μl of the diluted cDNA was then added to each 20-μl PCR mixture. The qRT-PCR was performed 2-step method. Each sample was carried out with three independent biological replicates and three technical replicates. Relative expression levels were calculated by the comparative 2^−ΔΔCT^ method using the expression of *actins* as the internal control.

### Statistical analyses

One-way analysis of variance (ANOVA) with the least significant difference (LSD) was performed for all data using SPSS 20.0 software (BM Corp, Armonk, NY, USA). Each experiment was repeated three times independently and the represent significance was considered to *P* < 0.05. Origin 8.1 (https://www.originlab.com/) and R3.5.1 (https://www.r-project.org/) were used to display the data obtained from the experiments.

## Supplementary information

**Additional file 1: Table S1.** Primers used in qRT-PCR. **Figure S1.** Graphical Abstract.

## Data Availability

The data generated and analysed in this study are available from the corresponding author on reasonable request. And all the sequencing data generated in this study have been deposited in the NCBI with the link of http://www.ncbi.nlm.nih.gov/bioproject/578101, under the accession number SAMN13049031, the Temporary Submission ID SUB643610 and the BioProject ID PRJNA578101.

## Abbreviations

ABA: Abscisic acid
ATP: Adenosine triphosphate
Aux: Auxin
BR: Brassinosteroid
bZIP: Basic leucine-zipper
CBF: C-Repeat binding factor
CI: Chilling injury
CK: Cytokinin
DEG: Differentially expressed gene
EL: Electrolyte leakage
ET: Ethylene
FDR: False discovery rate
FPKM: Fragments per kilobase million
GA: Gibberellin
GO: Gene ontology
GSH: Glutathione
GST: Glutathione s-transferase
HCT: Hydroxycinnamoyl-CoA shikimate
IAA: Indole acetic acid
JA: Jasmonic acid
LAR: Leuco anthocyanidin reductase
LOX: Lipoxygenase
LT: Low temperature
MDA: Malondialdehyde
NR: Non-redundant protein sequences
PAL: Phenylalanine ammonia lyase
Pfam: Protein family
PYL: Pyrabactin resistance 1-like
PYR: Pyrabactin resistance
ROS: Reactive oxygen species
RT: Room temperature
SA: Salicylic acid
TF: Transcription factors
TR: Transcriptional regulator
