# Supplementary material for: Transcriptome analysis of postharvest blueberries (Vaccinium corymbosum ‘Duke’) in response to cold stress
Source: BMC Plant Biol. 2020 Feb 19;20:80. doi: 10.1186/s12870-020-2281-1 (PMC7031921; doi:10.1186/s12870-020-2281-1)
Supplement: Supplementary file 1 — Additional file 1: Table S1. Primers used in qRT-PCR. Figure S1. Graphical Abstract. [file 12870_2020_2281_MOESM1_ESM.docx]

Additional file 1 (**TableS1** Primers used in qRT-PCR; **FigureS1** Graphical Abstract)

**TableS1** Primers used in qRT-PCR

|  | Name | Gene Number | Sense Primer (5′-3′)  Forward/ Reverse primer (5′-3′) | Anti Primer (3′-5′) |
| --- | --- | --- | --- | --- |
|  | Actin |  | ACTACCATCCACTCTATCACCG | AACACCTTACCAACAGCCTTG |
| 1 | allB | c123543.graphc0 | AGACCCTCCAAAGCACTTCTA | GATTTCCTTCAGGCACCAA |
| 2 | HIBCH | c120356.graphc0 | GCACGAGTGTCCTTCACAG | TTTGCTGCCTCACTTTCC |
| 3 | BCKDHA | c122233.graphc0 | AAACCACGCCACAATAGGA | AAGCACAGAGGCAAGGGA |
| 4 | gdhA | c126860.graphc0 | GGAAGACAGCAGTAGCCAACA | TGAGCAGCCCAAGAACCC |
| 5 | ALDH18A1 | c122210.graphc0 | CACAAGCCTTCCCATCAA | TTACATCAGGAGCCGTCAG |
| 6 | PAO2/3/4 | c123304.graphc0 | AGGTCATTCCGTCCTTGTTTA | AGCCGCCATCAGTCCAGT |
| 7 | PRPS | c121781.graphc0 | AGCAACATTGTGCCCGTAA | GCCAAACTTGTAGCGAACCT |
| 8 | G6PD | c129827.graphc0 | TGCTGCGGCTTTGTTTAT | GTGGCAAGATCACTGTTATGG |
| 9 | IDH1 | c123222.graphc0 | CGAAGTCCTAACGGCACA | TTCCACTGGCAAATCACC |
| 10 | GPX | c112498.graphc0 | AATCTGTCCACGAGTTCACC | TTCCACTTTATGCTGTCCC |
| 11 | GST | c112252.graphc0 | TGTGAGTCCCTCGTCATTGTCC | TGCTGCCTTCTTGGCTTCC |
| 12 | GST | c119253.graphc0 | AAAGGTAGAATGGGATCAGGG | CTTGGGAGGCAATTATGGA |
| 13 | GST | c102135.graphc0 | AAGGATCAGCAGGCAACAGA | CTCGGCGACAAGCCATAC |
| 14 | GST | c105671.graphc0 | GAAGGGAATCCAATATGAAATG | CAGGGTTGTGCTCAAGAAGTA |
| 15 | GST | c105671.graphc1 | TATGAAATGATACCGCAAGAAC | TTAGCCCAGAAACGAGCC |
| 16 | GST | c122443.graphc0 | GAAACTGAAGGGAATCCAATA | GAACTTAGCCCAGAAACGAG |
| 17 | CYP85A2 | c115136.graphc0 | AAAGAGCAAGGTTTGGGAGT | GGGAAGGTTTATGGGAAGTGA |
| 18 | CYP90A1 | c112010.graphc0 | CGAGCGTCCTTGAAGTGGT | GATTTCTTGGTGGCGTTGC |
| 19 | crtZ | c99806.graphc0 | ATAAGGAAGCGTGCCAGAGCG | GAATGGAGTTTTGGGCGAGATG |
| 20 | NCED | c126206.graphc0 | TCCGATAAATGAGGCGACAAT | GTTCACCATCCCTGCTTCCA |
| 21 | ABA2 | c125901.graphc0 | CAGCATTAGCCACATCATCAG | TGTTTCGGGACTCACCAAG |
| 22 | CYP707A1 | c120652.graphc1 | AGAAATGTATTGGGTTTAGGTG | TTATCTTATCCCGAAAGGGT |
| 23 | CKX | c111548.graphc0 | TTGTCAAATGCTGGAATTAGTG | TTGCCTTGGAGGGAGATG |
| 24 | LAR | c110095.graphc0 | GGCAGCACCCTTTCTCCT | GCCCTCAATAAATCCGTTCA |
| 25 | LAR | c119207.graphc1 | TATTTCAGCCGTAGGTGGTG | GTTTAGATAATTGCATGGTGGTC |
| 26 | HCT | c116187.graphc0 | TCACAATCCCACCCTTCATCG | GGAGCGGGCTGGTATTCTACG |
| 27 | IAA | c128076.graphc2 | GGCGGAATCCAGAACAAT | ATCTCGACGCATCATAAAGTG |
| 28 | IAA | c122133.graphc1 | GGCGGAATCCAGAACAAT | ATCTCGACGCATCATAAAGTG |
| 29 | GH3 | c111137.graphc1 | TGCTTCGTATTGGTGCCG | GTTGTGGGTTAGGTTTCAGGAT |
| 30 | TIR1 | c113524.graphc0 | TTTCGCAACCAGACAGCAC | GTTCCAGTACATTGGCACCC |
| 31 | SAUR | c102439.graphc0 | TTTGTTGAAGGAGGCAGAGG | GGCAAGGGATGGTGATGG |
| 32 | AHK2/3/4 | c122606.graphc0 | GGTTCTCACTGCTCCTTTCA | CCTCCACTTTGGCTATTCG |
| 33 | ARR-B | c107755.graphc0 | ACCATCCATGTCAGGCATAT | CCAAGTTACAACGACCAATC |
| 34 | ARR-A | c107605.graphc0 | TTCCCCAAACAGTCATCA | TCCTTCTTCCAAGCATCT |
| 35 | ARR-A | c116928.graphc0 | TTAAACAGGAATTGGAGGTG | CTGCTTATTCTTGAAGGGAC |
| 36 | PYL | c121430.graphc1 | AGTTGATGTTAAATCTGGGCTTCC | TGGGCTCGGTCCTGTCTTG |
| 37 | BSK | c122496.graphc0 | CACGACCTAAATCCTTACAGAGT | GCGAGATACCCATCAAGACAT |
| 38 | BSK | c129050.graphc0 | CCCCATCACTTGCTGCTCT | CAAATCCGATGTCTTCCACC |
| 39 | TGA | c126145.graphc0 | CAGAAGCATCTCCTCCAACA | AACCAACTCCCTTCCTCTTT |
| 40 | PR1 | c119719.graphc1 | CAGGTGGTATGGAGGAAGTCG | CCATTGTTGCACCGAGCC |


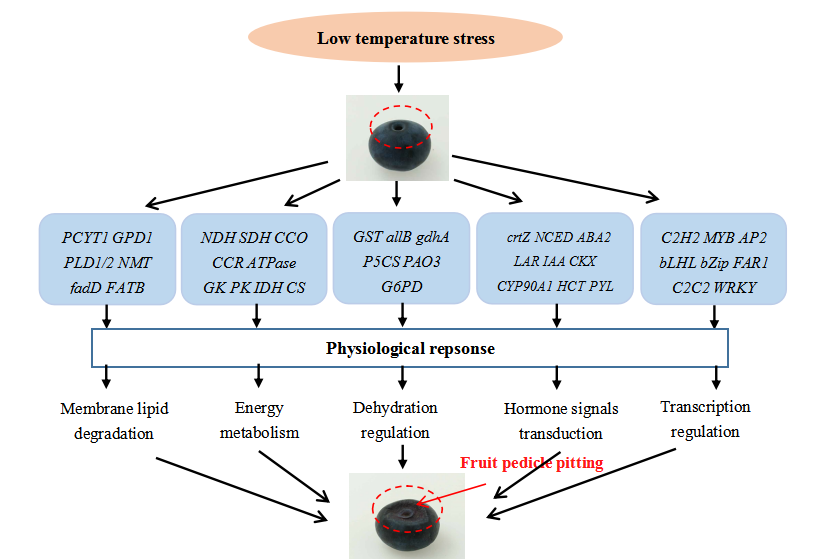
**FigureS1** Graphical Abstract
